# Extracellular and intracellular cleavages of proBDNF required at two distinct stages of late-phase LTP

**DOI:** 10.1038/npjscilearn.2016.3

**Published:** 2016-05-11

**Authors:** Petti T Pang, Guhan Nagappan, Wei Guo, Bai Lu

**Affiliations:** 1 National Institute of Child Health and Human Development, Bethesda, MD, USA; 2 Sanofi-Genzyme, Framingham, MA, USA; 3 GlaxoSmithKline, R&D China, Shanghai, China; 4 School of Medicine, Tsinghua Univ., Beijing, China

## Abstract

Although late-phase long-term potentiation (L-LTP) is implicated in long-term memory, its molecular mechanisms are largely unknown. Here we provide evidence that L-LTP can be divided into two stages: an induction stage (I) and a maintenance stage (II). Both stages require mature brain-derived neurotrophic factor (mBDNF), but involve distinct underlying mechanisms. Stage I requires secretion of existing proBDNF followed by extracellular cleavage by tPA/plasmin. Stage II depends on newly synthesized BDNF. Surprisingly, mBDNF at stage II is derived from intracellular cleavage of proBDNF by furin/PC1. Moreover, stage I involves BDNF-TrkB signaling mainly through MAP kinase, whereas all three signaling pathways (phospholipase C-γ, PI3 kinase, and MAP kinase) are required for the maintenance of L-LTP at stage II. These results reveal the molecular basis for two temporally distinct stages in L-LTP, and provide insights on how BDNF modulates this long-lasting synaptic alternation at two critical time windows.

## Introduction

Long-term memory is believed to be mediated by long-lasting, protein synthesis-dependent changes in synaptic efficacy. The best-studied cellular model is the late-phase long-term potentiation (L-LTP) in the hippocampus. L-LTP requires gene transcription and new protein synthesis, and is accompanied by dendritic growth and synaptic remodeling.^1^ Although tetanic stimulation used to induce L-LTP trigger the expression of many proteins, the specific protein synthesis product(s) responsible for the induction and maintenance of L-LTP remain to be established. Although several molecules were reported to be involved in L-LTP, such as dopamine D1/D5 receptors, mGluR and PKA,^2–4^ the most important candidate is brain-derived neurotrophic factor (BDNF), which is a major secretory neurotrophic factor in the brain.^5–7^ L-LTP inducing stimuli elicits an increase in hippocampal BDNF mRNA, with a time course well correlated with L-LTP expression and the formation of long-term memory.^8–15^ Inhibition of BDNF signaling significantly impairs L-LTP as well as long-term memory as assessed by several behavior tests.^16–18^ In addition, BDNF has been identified as one of the few CREB-dependent proteins critical for the maintenance of L-LTP. In VP16-CREB (constitutively active CREB) over-expressing mice, L-LTP can be induced by weak tetanus and is no longer dependent on protein synthesis.^19^ Furthermore, when protein synthesis is blocked during the entire course of L-LTP, application of BDNF completely reverses the L-LTP deficit.^5^ These results suggest that BDNF is at least one of the key protein synthesis products mediating L-LTP.

Similar to other neurotrophins, BDNF is first synthesized as a precursor termed proBDNF, which is then converted to mature BDNF (mBDNF) through the proteolytic removal of the N-terminal fragment by specific protease(s).^20^ Interaction of mature neurotrophins with Trk receptors leads to cell survival, whereas binding of pro-neurotrophins (proBDNF, proNGF) to p75 NGF receptor (p75^NTR^) leads to apoptosis.^21,22^ Pro-NGFs were initially shown to be processed by intracellular proteases including the serine protease furin (FIN) in the trans-Golgi network and the prohormone convertases (PC1/3) in the secretory granules.^23,24^ It has also been demonstrated extracellular cleavage of proNGF and proBDNF by matrix metalloproteinases (MMP3 or MMP7) and the serine protease plasmin.^21,25^

Activation of plasmin, which is initially produced as an inactive zymogen, plasminogen, requires cleavage by tissue plasminogen activator (tPA).^26^ Given that tPA is a secreted protease selectively involved in L-LTP,^27,28^ it has been hypothesized that extracellular cleavage of proBDNF by tPA/plasmin may somehow be involved in synaptic modulation by BDNF.^20^ Indeed, we have shown that tPA, by activating plasminogen, converts proBDNF to mBDNF in the hippocampus, and such conversion is critical for L-LTP expression.^5^ This work has provided a mechanistic link between tPA and BDNF in L-LTP, and revealed a physiological role of extracellular cleavage of proBDNF. We also have shown that proBDNF, if not processed, selectively enhances NMDA receptor-dependent long-term depression by activating its preferred receptor p75^NTR^.^29^ Thus, parallel to the roles of pro/mature NGFs in mediating cell death/survival, proBDNF and mBDNF also elicit opposite effects on long-term synaptic plasticity.^25,30,31^ Recent work has demonstrated that high-frequency neuronal activity induced the secretion of tPA in extracellular proBDNF to mBDNF conversion.^32^ Interestingly, this conversion has been shown to mediate activity-dependent synaptic competition during neuromuscular synapse formation.^33,34^ Cleavage of pro-NGFs by intracellular or extracellular proteases has now emerged as an important mechanism in controlling the direction of NGF regulation.

The requirement for proBDNF→mBDNF conversion in L-LTP provides a unique opportunity to investigate the mechanisms underlying L-LTP, which is largely unknown thus far. Is tPA/plasmin the only enzyme system involved in the cleavage of proBDNF? When does tPA/plasmin cleavage of proBDNF take place during the course of L-LTP? During our study of BDNF regulation, we unexpectedly found that L-LTP in hippocampal CA1 synapses can be divided into two temporally distinct stages: an induction (I) and a maintenance (II) stage, based on their sensitivity to the inhibition of tPA/plasmin and protein synthesis inhibitors. We show that both stages require BDNF, but each stage engages distinct underlying mechanisms. Conversion of secreted proBDNF to mBDNF by tPA/plasmin protease system is required at stage I, but not stage II. In contrast, mBDNF might function as a product of activity-dependent translation, responsible for the maintenance of L-LTP at stage II, but not stage I. By using a membrane impermeable inhibitor for tPA and a membrane permeable inhibitor for FIN/PC1, we demonstrated that extracellular cleavage of proBDNF is required for stage I, whereas intracellular cleavage is required for stage II. These results provide important insights into the molecular mechanisms underlying L-LTP and long-term memory.

## Results

### BDNF is required at both induction and maintenance stages of L-LTP

**Figure 1:**
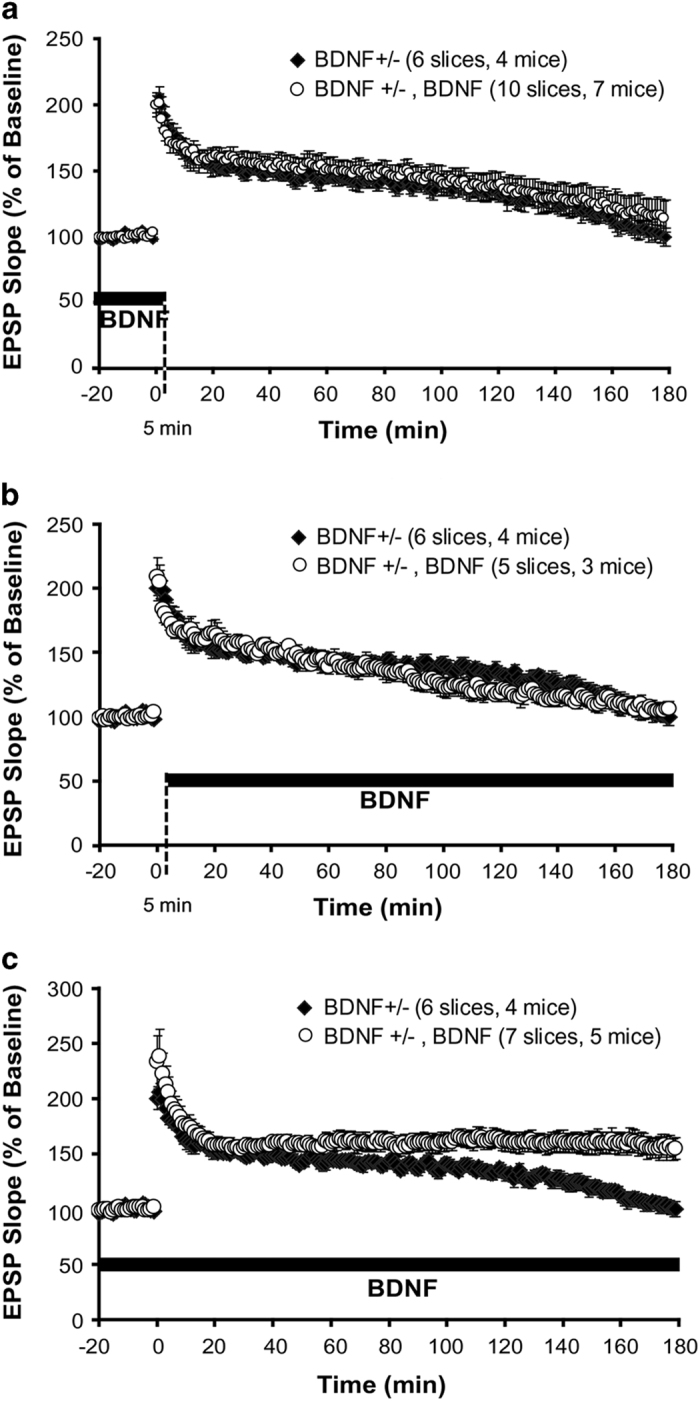
BDNF is required at both induction and maintenance stages of late-phase LTP (L-LTP). In this and other figures involve the use of various mutant mice, field EPSPs were recorded in CA1 area of hippocampal slices. L-LTP was induced by a ‘12-theta burst’ stimulation (l-TBS). Application of various drugs is indicated by horizontal bars. (**a**) BDNF fails to rescue L-LTP in BDNF+/− slice when it was applied at stage I. In this and all other relevant figures, BDNF was perfused from 60 min before to 5 min after l-TBS (indicated by a dotted line), but only 20 min of BDNF treatment before l-TBS is shown. (**b**) BDNF applied at stage II (5 min after l-TBS until the end of the recording, indicated by a dotted line) also could not rescue L-LTP on BDNF+/− slices. (**c**) BDNF rescued L-LTP when it was applied at both stages (for the entire period of recording). BDNF, brain-derived neurotrophic factor; EPSP, excitatory postsynaptic potential; LTP, long-term potentiation; tPA, tissue plasminogen activator.

Field recordings were performed in hippocampal slices from wild-type and conventional BDNF heterozygous mutant (BDNF+/−) mice. L-LTP was induced by applying ‘12-theta burst’ stimulation (or long TBS, l-TBS in brief) to Schaffer collateral afferents of CA1 synapses.^5^ Consistent with previous studies,^5,35^ a severe impairment in L-LTP was observed in slices derived from the BDNF+/− mice (Figure 1a). To determine whether there is a specific time window in L-LTP that is sensitive to BDNF regulation, we applied exogenous BDNF (200 ng/ml) to BDNF+/− (control) slices at two different stages during L-LTP. Application of BDNF 60 min before to 5 min after l-TBS failed to rescue L-LTP in BDNF+/− slices (Figure 1a). Field excitatory postsynaptic potential (fEPSP) measured 3 h after l-TBS in control 101.8±8.6% of baseline, but 115.5±12.7% in ‘+ BDNF’ group (Student’s *t*-test, *P*=0.408). Similarly, BDNF applied 5 min after l-TBS until the end of the recording did not rescue L-LTP in BDNF+/− slices (Figure 1b, control, 101.8±8.6%;+BDNF, 105.1±5.8%, *P*=0.788). However, perfusion of BDNF throughout the entire experiment completely rescued L-LTP in BDNF+/− mice (Figure 1c, control, 101.8±8.6%;+BDNF, 155.7±9.7%, *P*<0.005). Acute application of BDNF to wild-type hippocampal slices has also been shown to potentiate basal synaptic transmission.^36,37^ This requires high concentration of BDNF perfused at a fast rate (4 ml/min).^38^ Application of BDNF at a lower perfusion rate (⩽2 ml/min) in our hands did not elicit a change in basal synaptic transmission (data now shown). Similar results were obtained by others.^39^

Consistent with our findings, application of the TrkB inhibitor 1NMPP1 to the TrkB(F616A) knock-in mice around the time of TBS^40^ prevented L-LTP, implying that BDNF is required at stage I of L-LTP. Moreover, application of the BDNF scavenger TrkB-Fc 90 min after TBS reversed L-LTP, indicating that BDNF is also required at stage II.^41^ Taken together, these results suggest that L-LTP can be divided into two BDNF-dependent stages: the induction stage (or stage I) that spans the period before l-TBS to 15–30 min after l-TBS, and the maintenance stage (or stage II) that covers the entire period after stage I.

### Cleavage of proBDNF by tPA/plasmin is required at stage I, but not stage II

**Figure 2:**
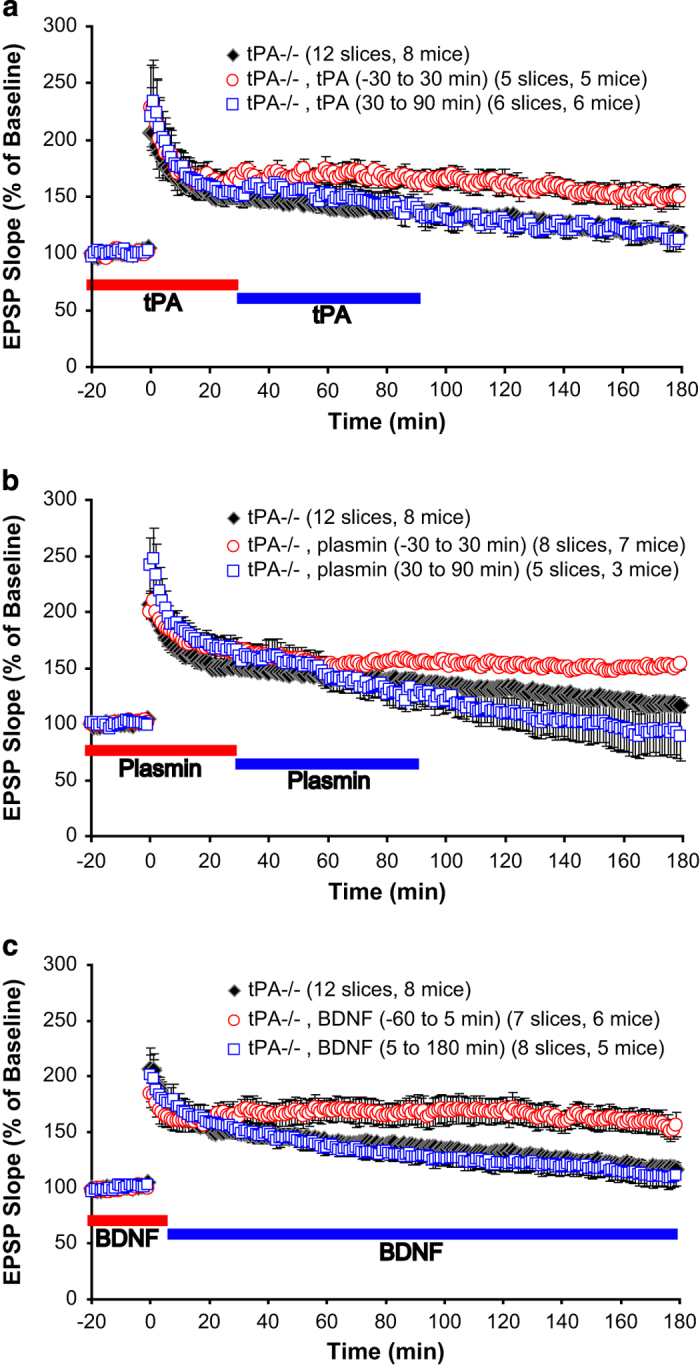
Cleavage of proBDNF by tPA/plasmin is required at stage I, but not stage II. Various drugs were applied to hippocampal slices derived from the tPA homozygous (−/−) mice at stage as indicated. (**a**) tPA rescued L-LTP deficit in tPA−/− slices only when it was applied at stage I (from 30 min before 30 min after l-TBS), but not stage II (from 30–90 min after l-TBS). (**b**) Plasmin also rescued L-LTP deficit when applied at stage I, but not stage II. (**c**) Similarly, BDNF rescued L-LTP deficit when applied at stage I, but not stage II. Note that due to its sticky nature, BDNF was applied and washed away earlier than tPA and plasmin (−60 min at stage I and 5 min at stage II). At stage II, leaving BDNF till the end of the recording still did not rescue L-LTP deficit. BDNF, brain-derived neurotrophic factor; L-LTP, late-phase long-term potentiation; tPA, tissue plasminogen activator.

Our previous study showed that proteolytic cleavage of proBDNF by the tPA/plasmin system is critical for L-LTP.^5^ To determine the specific stage in which tPA/plasmin is required for L-LTP, exogenous tPA was applied to tPA−/− slices at stage I or II to see whether it could rescue L-LTP in tPA −/− animals. Perfusion of tPA (500 ng/ml) at stage I (from 30 min before to 30 min after l-TBS) completely rescued L-LTP (Figure 2a, tPA−/−, 110.4±6.8%; tPA−/−+tPA, 150.3±9.0%, *P*<0.05), whereas application of tPA at stage II (30–90 min after l-TBS) failed to restore L-LTP in tPA−/− slices (Figure 2a, tPA−/−, 110.4±6.8%; tPA−/−+tPA, 113.4±12.0%, *P*=0.840). These results suggest that presence of tPA in the induction stage is sufficient to maintain L-LTP expression. Consistent with this notion, tPA, when applied during a single train tetanus that normally only induces E-LTP, caused the expression of L-LTP.^28^

Given that tPA cleaves plasminogen to form plasmin, we predicted that application of plasmin at the specific stage that is tPA sensitive would rescue L-LTP in tPA−/− mice. As shown in Figure 2c, perfusion of plasmin (100 nM) at stage I effectively rescued L-LTP in tPA−/− slices (Figure 2b, tPA−/−, 110.4±6.8%; tPA−/−+plasmin, 149.7±5.0%, *P*<0.005). In contrast, perfusion of plasmin at stage II failed to rescue L-LTP (Figure 2b, tPA−/−, 110.4±6.8%; tPA−/−+plasmin, 105.1±23.5%, *P*=0.777). These results suggest that activation of plasmin by tPA is involved in stage I but not stage II of L-LTP.

Plasmin is known to convert proBDNF to mBDNF.^21,42^ Thus, we hypothesized that the pathway tPA→plasmin→mBDNF^5^ is involved in stage I, but not stage II, of L-LTP. To test this idea, we applied BDNF at stage I or II to tPA−/− hippocampal slices. As BDNF is a sticky molecule that requires longer time to perfuse into and wash away from the slices,^43^ we started applying BDNF to slices 60 min before l-TBS and washing it away 5 min after l-TBS at stage I. At stage II, BDNF was applied 5 min after l-TBS until the end of the recording. Application of BDNF (200 ng/ml) to the tPA−/− slices at stage I completely reversed the L-LTP deficit (Figure 2c, tPA−/−, 110.4±6.8%; tPA−/−+BDNF, 154.0±10.0%, *P*<0.005). In contrast, application of BDNF at stage II had no effect (Figure 2c, tPA−/−, 110.4±6.8%; tPA−/−+BDNF, 109.8±9.6%, *P*=0.960). Similar experiments were performed using plasminogen−/− animals. We found that application of BDNF at stage I, but not stage II, reversed the L-LTP deficit in plasminogen−/− mice (data not shown). Two conclusions can be drawn from these results. First, tPA/plasmin is critical for stage I, but not stage II, of L-LTP. Second, BDNF is required in the tPA/plasmin pathway during stage I of L-LTP.

### BDNF sustains protein synthesis-dependent L-LTP at stage II, but not stage I

**Figure 3:**
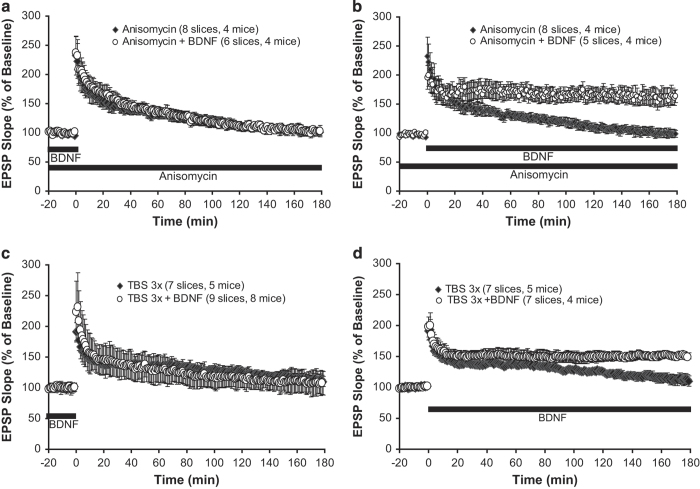
BDNF sustains protein synthesis-dependent L-LTP at stage II, but not stage I. BDNF was applied the same ways as in Figure 2c. (**a** and **b**) The protein synthesis inhibitor anisomycin (40 μmol/l) was applied as indicated by the bars to impair L-LTP in wild-type slices. BDNF restored L-LTP impairment only when applied at stage II, but not stage I. (**c** and **d**) Similarly, BDNF converted E-LTP (induced by TBS 3×) to L-LTP in wild-type slices only when it was applied at stage II but not in stage I. BDNF, brain-derived neurotrophic factor; L-LTP, late-phase long-term potentiation; tPA, tissue plasminogen activator.

A key feature of L-LTP is its dependency on new protein synthesis. Application of the protein synthesis inhibitor anisomycin (40 μM) throughout the recording completely prevented L-LTP (Figure 3a). Perfusion of BDNF 60 min before to 5 min after l-TBS could not rescue L-LTP (Figure 3a, anisomycin, 101.4±6.6%; anisomycin+BDNF, 102.6±7.5%, *P*=0.885), while BDNF perfused 5-180 min after l-TBS reversed the L-LTP impairment by anisomycin (Figure 3b, anisomycin, 101.4±6.6%; anisomycin+BDNF, 164.5±13.7%, *P*<0.005). These results suggest that in the absence of new protein synthesis, BDNF could reverse the L-LTP deficit at stage II, but not stage I. Consistent with this notion, Santi *et al.* ^39^ showed that endocytosed exogenous BDNF (through pre-incubation) could rescue the L-LTP deficit when endogenous BDNF synthesis was inhibited by anisomycin, although they did not establish which stage BDNF acts to counter the anisomycin effect. To further investigate the role of BDNF in L-LTP maintenance, we used the ‘3-thetaburst stimulation’ (short TBS, or s-TBS in brief), which is known to induce only E-LTP but not L-LTP (Pang *et al.* ^5^). Application of BDNF at stage II converted E-LTP to L-LTP (Figure 3d, TBS 3×, 112.5±8.6%; TBS 3×+BDNF, 151.1±5.0%, *P*<0.005). In contrast, application of BDNF at the stage I failed to do the same (Figure 3c, TBS 3×, 112.5±8.6%; TBS 3×+BDNF, 107.7±108.5%, *P*=0.638). Thus, BDNF appears to work at stage II to support the protein synthesis-dependent expression of L-LTP.

### Signaling mechanisms underlying BDNF regulation at stage I and stage II

**Figure 4:**
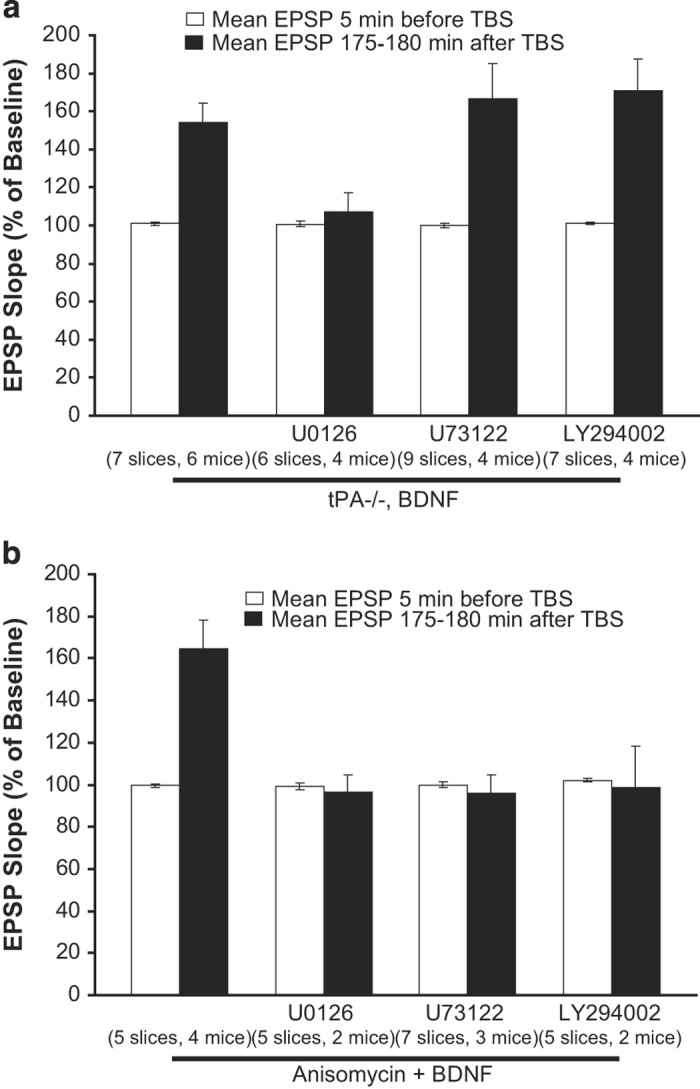
Signaling mechanisms underlying BDNF regulation at stage I and stage II. (**a**) MAP kinase, but not PLC-γ orPI3K, is required for BDNF regulation of L-LTP at stage I. Hippocampal slices from tPA−/− mice were perfused with BDNF, together with indicated drugs, at stage I. BDNF applied at stage I rescued the L-LTP deficit in tPA−/− slices, but simultaneous application of the MAPK inhibitor U0126 reversed the BDNF effect. Neither inhibition of PLC-γ by U73122 nor inhibition of PI3K by LY294002 altered the rescuing effect of BDNF on L-LTP. (**b**) All three signaling pathways are required for BDNF regulation at stage II. Wild-type slices were perfused with anisomycin throughout the recording to block protein synthesis. BDNF, together with indicated drugs, was applied at stage II as. Inhibition of MAPK by U0126, PLC-γ by U73122, or PI3K by LY294002 (**c**) blocked the rescuing of BDNF on L-LTP. BDNF, brain-derived neurotrophic factor; L-LTP, late-phase long-term potentiation; tPA, tissue plasminogen activator.

We next investigated whether BDNF uses the same or different signaling mechanisms to regulate stage I and stage II of L-LTP. Specific inhibitors were individually applied to block one of the three signaling pathways downstream of TrkB tyrosine kinase activated by BDNF. For mechanisms involving stage I, we used tPA−/− slices. When the MAPK inhibitor U0126 (30 μM) was administered together with BDNF at stage I, the effect of BDNF in rescuing the L-LTP deficit in tPA−/− slices was markedly attenuated (Figure 4a). In tPA−/− slices treated with BDNF at stage I, fEPSP measured 3 h after l-TBS was 154.0±10.1%. In separate slices in which BDNF was applied together with U0126, the fEPSP at the same point was 107.4±10.21% (*P*<0.05). In contrast, co-application of the phospholipase C-gamma (PLC-γ) inhibitor U73122 (5 μM) with BDNF at stage I did not block the rescue of L-LTP by BDNF in tPA−/− slices (Figure 4a, tPA−/−+BDNF, 154.0±10.1%; tPA−/−+BDNF+U73122, 166.4±18.8%, *P*=0.569; Supplementary Figure 1). Similarly, the PI3K inhibitor LY294002 (30 μM) failed to reverse the effect of BDNF (Figure 4a, tPA−/−+BDNF, 154.0±10.1%; tPA−/−+BDNF+LY294002, 170.9±17.0%, *P*=0.410; Supplementary Figure 1). These results suggest that BDNF may signal through MAPK to regulate the induction stage of L-LTP.

For mechanisms involving stage II, we applied the protein synthesis inhibitor anisomycin throughout the experiment to block L-LTP. As shown before, application of BDNF from 5 min after the delivery of l-TBS until the end of the experiments completely rescued L-LTP. Similar to that in stage I, the MAPK inhibitor U0126 was able to attenuate the effect of BDNF in reversing the L-LTP deficit elicited by anisomycin (Figure 4b, anisomycin+BDNF, 164.5±13.7%; anisomycin+BDNF+U0126, 96.3±8.8%, *P*<0.001; Supplementary Figure 2). Unlike stage I, however, either the PLC-γ inhibitor U73122 or the PI3K inhibitor LY294002 reversed the effect of BDNF (Figure 4b). Thus, signaling pathways via MAPK, PLC-γ and PI3K are all required in the maintenance stage of L-LTP. Taken together, these results suggest that BDNF uses different signaling mechanisms to regulate stage I and II of L-LTP.

### Immunofluorescence detection of secretion and processing of BDNF

**Figure 5:**
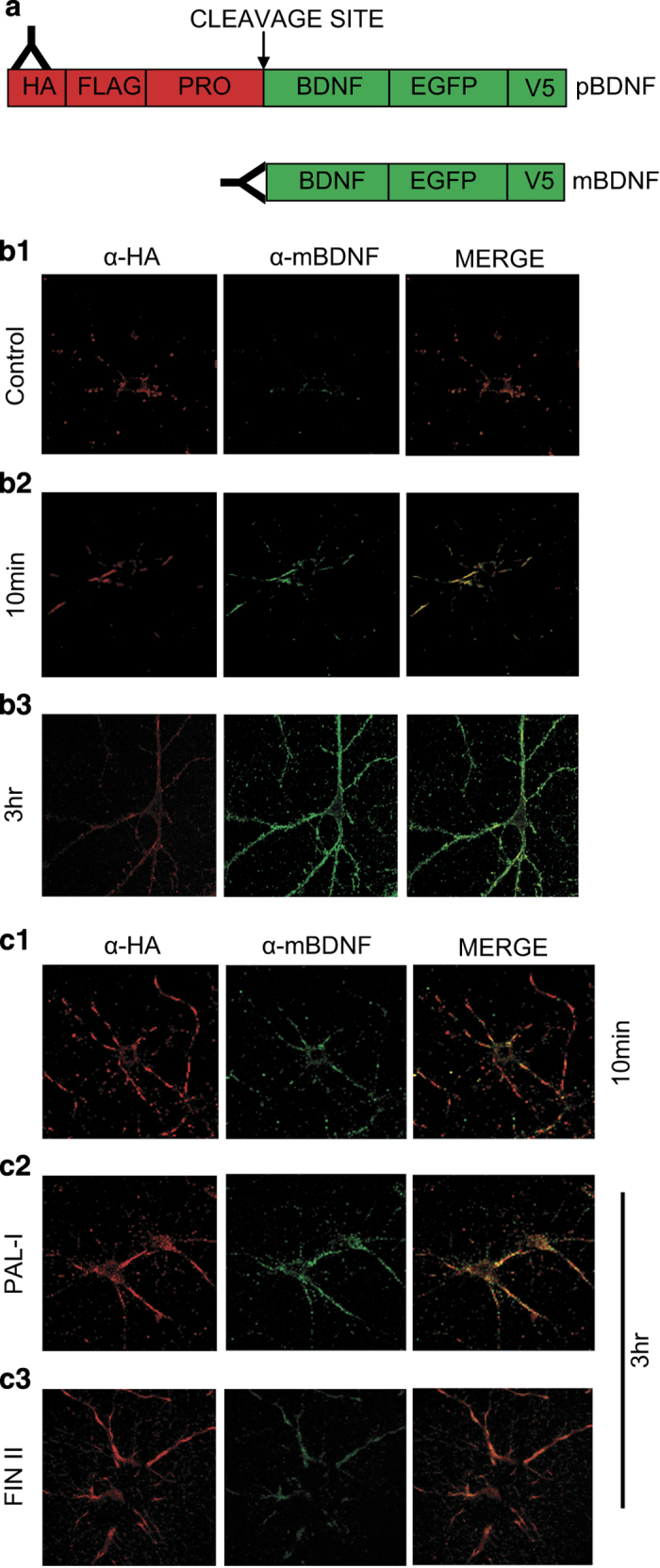
TBS increases surface staining of proBDNF at stage I and mBDNF at stage II. (**a**) A schematic diagram showing the detection of proBDNF by HA antibody (red) and mBDNF antibody (green), respectively. (**b1**) proBDNF and mBDNF on neuronal surface in control conditions (before electrical stimulation) by non-permeable immunofluorescence staining. (**b2**) Expression of predominantly proBDNF on neuronal surface at stage I. Cultured neurons were subjected to field TBS stimulation for 10 min and instantly fixed for non-permeable immunofluorescence staining by the HA and mBDNF antibodies. Note that the surface of hippocampal neurons expressed mostly proBDNF (red). (**b3**) Expression of predominantly mBDNF on neuronal surface at stage II. Neurons from sister cultures were stimulated by TBS for 10 min, 3 h later the cultures subjected to the same non-permeable immunofluorescence staining. Note that the surface of hippocampal neurons expressed mostly mBDNF (green). (**c**) Transfected hippocampal neurons (14 DIV) were treated for 12 h with either furin inhibitor II (FIN II, 13 μmol/l) to block intracellular BDNF cleavage by furin or PC1, or the PAI-1 (1 μg/ml) to inhibit tPA. The cultures were stimulated by field TBS in the medium containing the specified inhibitor for 10 min and fixed 3 h later. Immunofluorescence staining was performed under non-permeable conditions using anti-HA antibody for proBDNF (red) or anti-mBDNF antibody for mBDNF (green). (**c1**) Staining after 10 min TBS in control neurons. (**c2**) Staining after 3 h after TBS in neurons pre-treated with PAI-1. (**c3**) Staining 3 h after TBS in neurons pre-treated with FIN II. Note that FIN II, but not PAI-1, prevented the appearance of cell surface mBDNF 3 h after field stimulation. BDNF, brain-derived neurotrophic factor; HA, hemaglutinin; L-LTP, late-phase long-term potentiation; mBDNF, mature BDNF; PC1, prohormone convertase; tPA, tissue plasminogen activator.

The fact that application of recombinant BDNF could reverse the L-LTP deficit in tPA−/− slices at stage I (Figure 2) and a similar deficit in anisomycin-treated slices at stage II (Figure 3) indicates that both stages require mBDNF. The lack of effects of the tPA/plasmin system at stage II of L-LTP (Figure 2) prompted us to further investigate the enzyme systems involved in proBDNF→mBDNF conversion at this stage. Due to the difficulties in determining the specific isoforms (proBDNF versus mBDNF) at synaptic clefts in hippocampal slices during stage I and stage II, we turned to cultured hippocampal neurons (>14 days) transfected with BDNF tagged with hemaglutinin (HA) at the N-terminal of proBDNF. Anti-HA antibody was used to detect proBDNF (Figure 5a). We also developed an antibody that recognizes selectively the cleaved N terminus of the mBDNF,^32^ based on the idea that cleavage of proBDNF will generate a previously unexposed epitope (Figure 5a). Western blot analysis indicated that this antibody recognized only mBDNF, but not proBDNF or the pro-fragment (pro-domain of BDNF fused to glutathione-S-transferase at the N terminus; Supplementary Figure 3). BDNF is often attached to its cell surface receptors after its secretion. We, therefore, performed immunofluorescence staining under non-permeable conditions to detect cell surface-bound BDNF. Indeed, double staining using an anti-V5 antibody (detect both proBDNF and mBDNF) and an antibody against the extracellular domain of p75NTR showed co-localization of cell surface BDNF with p75NTR (Supplementary Figure 4A). Surface BDNF was associated with surface TrkB (both full length and truncated), detected by an antibody against extracellular domain of TrkB (Supplementary Figure 4B). There was very little ‘free’ BDNF (not associated with receptors) on the cell surface of neurons.

The tight association of secreted BDNF with its receptors on cell surface, together with the rapid turnover of the receptors,^20,44^ offered a unique opportunity to study the steady-state of BDNF secretion in a given time window. We used the anti-HA (red) and anti-mBDNF (green) antibodies that could specifically label proBDNF and mBDNF, respectively. Field electrical stimulation (l-TBS for 10 min) was applied to the transfected neurons to induce BDNF secretion.^44^ Before electrical stimulation, there was few mBDNF puncta on the surface of these neurons, mostly on the cell body region (Figure 5b). After 10 min of 1-TBS, anti-HA antibody revealed many proBDNF-positive puncta on the surface of neuronal processes, with minimal mBDNF staining (Figure 5b). These results are consistent with the idea that within the first 10 min of 1-TBS (equivalent to stage I), proBDNF is the dominant BDNF isoform secreted and extracellular proteases are necessary to convert proBDNF to mBDNF during this stage of L-LTP. In contrast, the majority of surface bound BDNF measured 3 h after the 1-TBS was mBDNF (Figure 5b). As proBDNF can also be processed intracellularly by the protease FIN and PC1,^23,24^ the present result raised the possibility that proBDNF could be cleaved intracellularly before its secretion at stage II.

To test this idea, we inhibited intracellular or extracellular processing of proBDNF, and performed cell surface staining 3 h after l-TBS (equivalent to stage II). Wild-type hippocampal neurons were depolarized briefly (25 mM high K^+^ for 5 min) to remove the existing BDNF in the secretory granules. The cells were then incubated overnight with the cell permeable inhibitor FIN II to block intracellular cleavage by FIN and PC1.^45^ Alternatively, the cells were incubated with the membrane impermeable protein inhibitor PAI-1 to block extracellular cleavage by tPA/plasmin.^28^ Neurons were stimulated with l-TBS for 10 min, and processed for cell surface staining using proBDNF- and mBDNF-specific antibodies, either right at the end of 10 min or 3 h after. Unlike the control condition (Figure 5b), pretreatment with FIN II markedly reduced the amount of mBDNF (green) on the cell surface (Figure 5c). ProBDNF became the dominant isoform secreted at this stage. In contrast, pretreatment with PAI-1 did not significantly change the relative proportion of proBDNF and mBDNF, and mBDNF remained to be the major form on the cell surface (Figure 5c). Taken together, these results support the notion that hours after l-TBS stimulation, mBDNF is secreted after intracellular cleavage. This is very different from the stage shortly after l-TBS, when the secretion of proBDNF is followed by extracellular cleavage.

### Extracellular and intracellular cleavages of proBDNF are required at stage I and stage II of L-LTP, respectively

**Figure 6:**
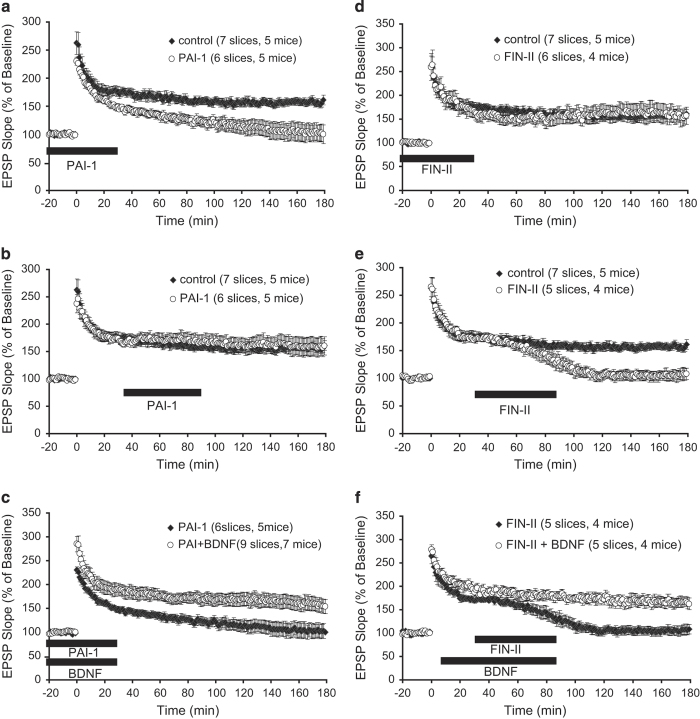
Extracellular cleavage of proBDNF by tPA/plasmin at stage I and intracellular cleavage by furin/PC1 at stage II. A membrane impermeable protein inhibitor for tPA, PAI-1, was applied to the wild-type slices at stage I or stage II to block extracellular tPA cleavage. (**a**) Application of PAI-1 at stage I impaired L-LTP. (**b**) Application of PAI-1 at stage II failed to impair L-LTP. (**c**) BDNF applied together with PAI-1 at stage I rescued the L-LTP deficit caused by PAI-1. (**d**) A membrane permeable peptide inhibitor for furin/PC1, FIN II, was applied to the wild-type slices at stage I or stage II to block intracellular cleavage by furin/PC1. Application of Fin II at stage I failed to impair L-LTP. (**e**) Application of FIN II at stage II effectively impaired L-LTP. (**f**) BDNF rescued the L-LTP impairment caused by application of FIN II at stage II. BDNF, brain-derived neurotrophic factor; L-LTP, late-phase long-term potentiation; PC1, prohormone convertase; tPA, tissue plasminogen activator.

We next investigated the role of intracellular and extracellular cleavage of proBDNF in different stages of L-LTP by applying the specific inhibitors to wild-type hippocampal slices. The cell-impermeable tPA inhibitor PAI-1 (1 μg/ml), when applied at stage I, blocked L-LTP (Figure 6a, control, 156.0.4±7.1%; PAI-1, 101.8±15.3%, *P*<0.05). In contrast, PAI-1 was completely ineffective when applied at stage II (Figure 6b, control, 156.0±7.1%; PAI-1, 159.1±16.9%, *P*=0.867). PAI-1 does not affect basal synaptic transmission.^28^ These results further support the notion that extracellular cleavage by tPA/plasmin is required at the induction stage of L-LTP, but not at the maintenance stage. To confirm that the impairment in L-LTP by PAI-1 is due to tPA-mediated proBDNF→mBDNF conversion, we perfused BDNF together with PAI-1 to the slices at stage I. Application of BDNF completely prevented the inhibitory effect of PAI-1 on L-LTP (Figure 6c, PAI-1, 101.8±15.3%; PAI-1+BDNF, 155.4±14.8%, *P*<0.05). Thus, the main function of tPA is to convert proBDNF to mBDNF during stage I of L-LTP.

To test the hypothesis that proBDNF is cleaved intracellularly and secreted as a mature form at stage II, the membrane permeable inhibitor FIN II was used to inhibit intracellular proteases FIN/PC1. Application of FIN II (50 μM) to hippocampal slices at stage I had no effect on L-LTP expression (Figure 6d, control, 156.0±7.1%; FIN II, 154.2±17.4%, *P*=0.926). However, when applied at stage II, this inhibitor markedly impaired L-LTP within a short period of time (Figure 6e, control, 156.0±7.1%; FIN II, 107.6±10.9%, *P*<0.05). Thus, intracellular cleavage of proBDNF by FIN/PC1 is required at the maintenance stage of L-LTP, but not at the induction stage. To confirm the impairment in L-LTP by the inhibitor results from the inhibition of proBDNF cleavage by FIN/PC1, BDNF was applied to the slices at 5 min after l-TBS and washed away with FIN II. Perfusion of BDNF completely reversed the inhibitory effect of the inhibitor on L-LTP (Figure 6f, FIN II, 107.6±10.9%; FIN II+BDNF, 165.6±11.4%, *P*<0.05). These results strongly suggest that at stage II, proBDNF is cleaved intracellularly by FIN/PC1, leading to the secretion of mBDNF to support L-LTP maintenance.

## Discussion

Compared with the extensive knowledge regarding E-LTP, relatively less is known about the molecular mechanisms underlying L-LTP. Thus far only two secreted proteins have been implicated in the process: the extracellular protease tPA and the NGF BDNF.^5^ The success of E-LTP research could be attributed, to certain extent, to the identification of temporally distinct processes within E-LTP such as induction, expression, and maintenance stages.^46^ Several different forms of L-LTP have been described, and previous work has established that BDNF is required for TBS-induced L-LTP, but not for L-LTP induced by multiple sets of tetanic stimulation.^35^ In this study, we have divided TBS-induced L-LTP into two stages based on its sensitivity to BDNF, and its proteolytic cleavage. Several early studies have hinted different windows of sensitivity to BDNF during L-LTP process. Given that BDNF promotes synaptic responses to tetanic stimulation,^47,48^ it is not surprising that treatment of hippocampal slices with the BDNF scavenger TrkB-IgG or the TrkB antibodies before and during the tetanic stimulation blocks L-LTP.^35^ Interestingly, application of TrkB-IgG 90 min after TBS could reverse L-LTP,^41^ suggesting that BDNF is also necessary in the maintenance phase of L-LTP. In the present study, BDNF+/− mice were shown to have a severe impairment in hippocampal L-LTP when BDNF was applied to the slices either before/during or after TBS stimulation, but perfusion of slices with BDNF during the entire time course completely reversed the L-LTP deficit. Our experiments have provided evidence that L-LTP requires BDNF in two temporally distinct stages, based on how proBDNF is cleaved: an induction stage (I) that depends on extracellular cleavage of secreted proBDNF by the tPA/plasmin system, and a maintenance stage (II) in which proBDNF is cleaved intracellularly by FIN/PC1 before its secretion. We have also demonstrated differential BDNF-TrkB signaling in these two different stages. These results underscore the physiological significance of proteolytic cleavage of proBDNF, and provide important insights into the cellular and molecular mechanisms of L-LTP. Division of L-LTP into multiple stages may facilitate mechanistic studies of L-LTP.

Upon binding to BDNF, the TrkB receptor undergoes autophosphorylation at several specific tyrosine residues with its intracellular domain.^49,50^ Of these, two are most interesting: pTyr816 forms a docking site for PLC-γ, and pTyr515 form a docking site for the adaptor protein Shc, which leads to the activation of both MAPK and PI3 kinase. Our study suggests that differential signaling mechanisms underlie BDNF modulation of L-LTP in two time windows: MAPK pathway is required in both stages, whereas PLC-γ and PI3 kinase are required only in the maintenance phase of L-LTP. BDNF has been shown to trigger the activation and nuclear translocation of MAPK in hippocampal neurons.^35,51^ Application of MAPK inhibitors either during or shortly after tetanus markedly attenuated L-LTP at CA1 synapses, suggesting that MAPK is critical for the full expression of BDNF-dependent forms of L-LTP.^6,52,53^ We now demonstrate that MAPK is required not only for stage I, but also for stage II. L-LTP stimuli activated the mTOR-p70 S6 kinase pathway in a PI3 kinase-dependent manner,^54^ and mTOR and PI3 kinase participated in forskolin-induced L-LTP.^55^ We now show that inhibition of PI3 kinase reversed the BDNF effect at stage II when protein synthesis was completely blocked. This result suggests that PI3 kinase mediates protein synthesis-independent effects of BDNF in stage II of L-LTP. Minichiello *et al.* ^56^ reported that L-LTP was selectively impaired in the knock-in mice in which the PLC-γ site of the TrkB receptor is mutated, but not in those in which the Shc site is mutated. It should be pointed out that the L-LTP-inducing protocol used in that study (3×100 Hz, 1 s, 5 min ISI) induces a form of L-LTP that is insensitive to BDNF, as shown by another study.^35^ It is possible that the strong tetanus used in the Minichiello’s study could trigger MAPK in the absence of the TrkB Shc site in a parallel signaling cascade that is yet to be identified. Nevertheless, the study using the PLC-γ site mutants points to the importance of PLC-γ in L-LTP. The present study further demonstrates that BDNF activation of PLC-γ is required at stage II, but not at stage I. In addition, the work of Yoshii *et al.* ^57,58^ also suggested that PI3 kinase and PLC-γ is required in transport and modification of PSD95, and it might explain the important roles of these two kinases in L-LTP.

Of great interest is the finding that proBDNF processed by different mechanisms during the two stages of L-LTP. At the stage I, high-frequency stimulation induces the secretion of proBDNF as well as tPA.^32^ This allows efficient processing of proBDNF extracellularly by the tPA/plasmin system to form mBDNF right at the synapses. Although direct evidence remains to be provided, it is possible that proBDNF at this stage is derived presynaptically from CA3 afferent terminals. Indeed, live fluorescence imaging demonstrated that the movement of BDNF–GFP (green fluorescent protein) fluorescent spots from cell bodies to axonal terminals in cortical neurons in an activity-dependent manner.^25,59,60^ Upon high-frequency synaptic stimulation of glutamatergic synapses, the BDNF–GFP or BDNF–QDs particles rapidly disappear, suggesting the secretion of BDNF from the synaptically localized secretory vesicles.^61–63^ In stage II, the existing BDNF protein may be exhausted after tetanus, and activity-dependent transcription/translation of new BDNF may be necessary to maintain a prolonged synaptic potentiation. Consistent with this idea, BDNF expression is increased hours after the application of L-LTP-inducing stimuli in hippocampal neurons.^8–11,19,64^ We show that at this stage, inhibition of intracellular but not extracellular processing of proBDNF blocked L-LTP, suggesting that the newly synthesized proBDNF may be cleaved intracellularly by FIN/PC1 before its secretion to support L-LTP maintenance. Intuitively, this mBDNF should be derived postsynaptically, possibly from the soma or dendrites of CA1 neurons. Optical imaging of a BDNF–GFP in transfected hippocampal neurons has demonstrated that BDNF could be packaged into secretory vesicles that are transported to somatodendritic compartment.^60–62^ These two different BDNF resources might be relevant to the study of the BDNF regulated translational control in dentate gyrus LTP consolidation, which was also divided into two stages.^65^

Mechanisms by which BDNF regulates L-LTP at stage I and stage II remain to be determined. It seems that BDNF at either stage I or stage II is necessary but neither alone is sufficient for L-LTP. Moreover, there seems to be a mechanistic connection between stage I and stage II. Recent studies from our own lab support the following model: BDNF secretion at stage I (a weak TBS is sufficient to induce) is to create a transient- and protein synthesis-independent synaptic tag – TrkB, while strong TBS triggers the synthesis of BDNF (plasticity-related protein) at stage II. The soma-derived BDNF is trafficked to the dendrites and captured by locally created TrkB tag at the synapses.^14,40^ Further investigations are necessary to validate this model.

In summary, our results strongly support the hypothesis that BDNF is required at two temporally distinct stages in L-LTP. At stage I, BDNF appears to be a target of the tPA/plasmin protease system that mediates the induction of L-LTP. At stage II, BDNF may act downstream of activity-dependent translation responsible for the maintenance of L-LTP. As there is evidence on the local secretion of BDNF at the presynaptic terminals shortly after tetanus^59^ and it has also been reported that tetanus induced the enhancement in BDNF mRNA expression at postsynaptic neuron with a time course well correlated with L-LTP expression and HFS-induced mBDNF secretion in distal dendrites,^9,32^ it is tempting to speculate that the requirement of BDNF at stage I involves presynaptic mechanism, while the requirement of BDNF at stage II is due to the involvement of postsynaptic mechanism. The experiments on the downstream mechanism of TrkB receptor also reveal the molecular basis for these two temporally distinct stages in L-LTP. Our study may help to understand how BDNF regulates L-LTP at different stages.

## Materials and Methods

### BDNF heterozygous mutant mice

BDNF mutant mice colony was raised from pairs of BDNF mutant mice with one allele of BDNF gene deleted (BDNF+/− mice) in C57BL/6 background.^66^ Plasminogen and tPA knockout mice, purchased from Jackson Laboratories (Bar Harbor, ME), were also in C57BL/6 background. tPA−/− mice were generated by homozygous crossing. Plasminogen−/− mice were generated by crossing plasminogen−/− males with plasminogen+/− females. Genotyping for tPA and plasminogen mice was performed according to the protocols provided by the Jackson Laboratories.

### Electrophysiological recording

Transverse hippocampal slices (400 μm) were prepared from wild-type and mutant mice (6–12 weeks old). The slices were maintained in an interface chamber for both recovery (2 h) and recording, and exposed to an artificial atmosphere of 95% O_2_/5% CO_2_, as described previously.^5^ Perfusion medium (artificial cerebrospinal fluid, 34 °C) contained (in mmol/l): NaCl 124, KCl 4.4, CaCl_2_ 2.5, MgCl_2_ 1.3, NaHCO_3_ 26.2, NaH_2_PO_4_ 1.0, glucose 10, ascorbic acid 2, pH 7.4. The perfusion rate was 2 ml/min. Recombinant BDNF (kindly provided by Regeneron Pharmaceuticals) was diluted in artificial cerebrospinal fluid containing 200 μg/ml bovine serum albumin and re-circulated in a volume of 20 ml. FEPSP were evoked in CA1 stratum radiatum by stimulating Schaffer collaterals with twisted bipolar nichrome electrodes and recorded with artificial cerebrospinal fluid-filled glass pipettes (<5 MΩ) using an Axoclamp-2B amplifier (Axon Instruments, Sunnyvale, CA, USA). Test stimuli consisted of monophasic 200 μs pulses of constant current were delivered by stimulus isolation units. Tetanic stimulation was applied after stable baseline was established for at least 20 min. L-LTP was induced by l-TBS, which contains 12 bursts, each with 4 pulses at 100 Hz and inter-burst interval of 200 ms. E-LTP was induced by s-TBS, which is essentially the same as l-TBS except 3 bursts instead of 12 bursts were used. Only slices exhibiting maximum fEPSP of 5–10 mV in amplitude without superimposed population spikes were used. Stimulus intensity was adjusted to evoke 40–50% of the maximum fEPSP, and basal synaptic transmission was monitored by alternating, low-frequency stimulation (every 30 s) via stimulating electrodes positioned on both sides of the recording electrode. Data are presented as mean±s.e.m.

### Nucleofection and surface labeling of BDNF in hippocampal neurons

Neuronal culture and neucleofection were carried out as described previously.^32^ The transfected neurons on cover slips were removed from the feeder layer into fresh 12-well plates overnight and then washed (3×) with Neurobasal medium containing 100 μg/ml bovine serum albumin. Following washes, the cover slips were left in 1.3 ml of neurobasal medium and subjected to field electric stimulation for 10 min using Master8 through bipolar platinum wires passing through the medium without touching the cells as described.^44^ TBS paradigm included multiple bursts applied at theta frequency with each burst consisting of four pulses at 100 Hz (individual pulses were 8 mV in amplitude with duration of 0.2 m; total of 2,500 bursts). The cover slips were then rinsed with phosphate-buffered saline (PBS) and fixed for 5 min with 4% paraformaldehyde in PBS (without calcium) containing 120 mmol/l sucrose followed by PBS containing 100 mmol/l glycine (5 min). The cover slips were blocked using 3% bovine serum albumin in PBS and then incubated with primary antibody (monoclonal anti-HA from covance at 1:200 and the custom made rabbit anti-mBDNF-specific antibody at 1:250) for 1 h followed by three washes for 5 min each with PBS. Antibodies were reinforced with anti-mouse (633) and anti-rabbit (488) Alexa fluor antibodies from Molecular Probes at 1:1,500 dilution, followed by washes with PBS and mounted on Mowiol 4–88 containing 2.5% anti-fade 1,4-diazobicyclo-[2.2.2]-octane (DABCO). Images were collected using Zeiss LSM 510 Meta confocal microscope (Göttingen, Germany) using 63X oil immersion objective and analyzed using LSM 510 software (München, Germany).

### Generation of mBDNF antibody

Seven amino acid peptide sequence corresponding to the amino terminus of the FIN cleaved end of the mBDNF (or B-peptide: HSDPARRC) was used for immunizing rabbits after conjugating to keyhole limpet hemocyanin (KLH) through a Cysteine at the C terminus. After three boost injections, the rabbit serum was collected and affinity purified on the agarose column conjugated to B-peptide. The affinity-purified antibody was then depleted on a column conjugated to the A+B peptide (Figure 4a), with the following sequence: CSMRVRRHSDPARR corresponding to the uncleaved proBDNF. The flow through of this procedure contains the antibody that specifically recognizes the cleaved end of the mBDNF, but not the uncleaved pBDNF.
