# Supplementary figures and images for: Extracellular and intracellular cleavages of proBDNF required at two distinct stages of late-phase LTP
Source: NPJ Sci Learn. 2016 May 11;1:16003. doi: 10.1038/npjscilearn.2016.3 (PMC6380376; doi:10.1038/npjscilearn.2016.3)

**A**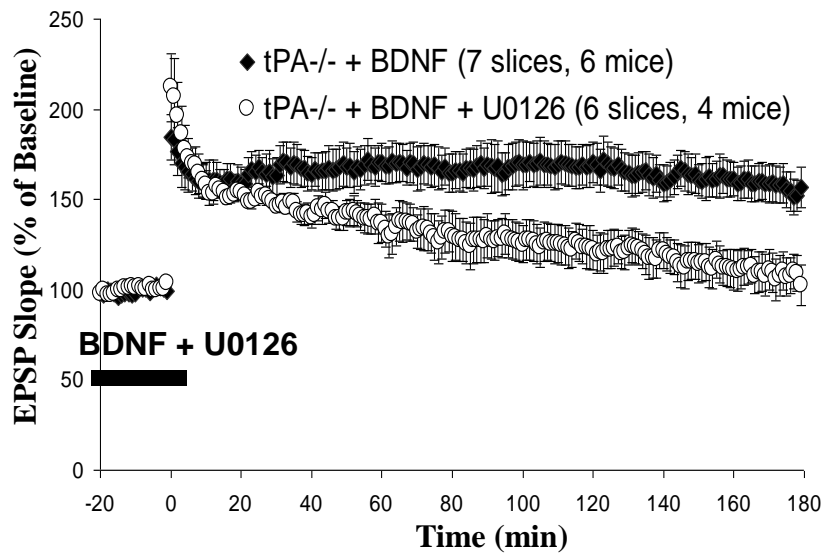**B**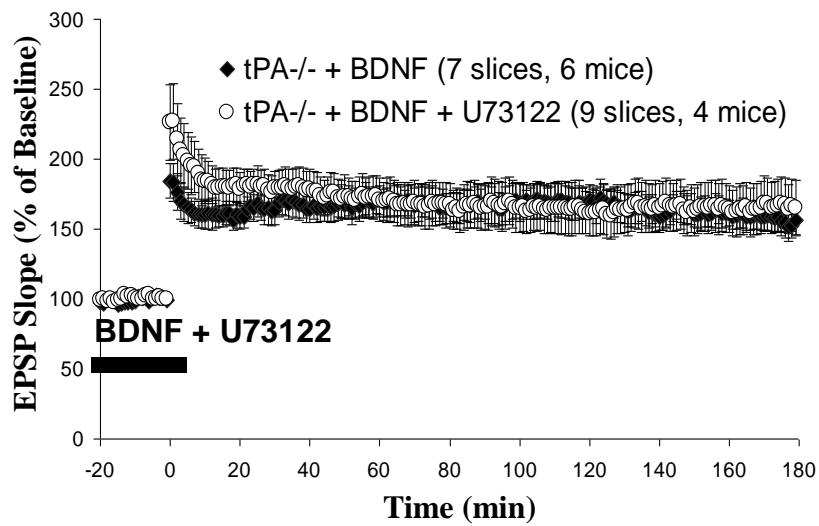**C**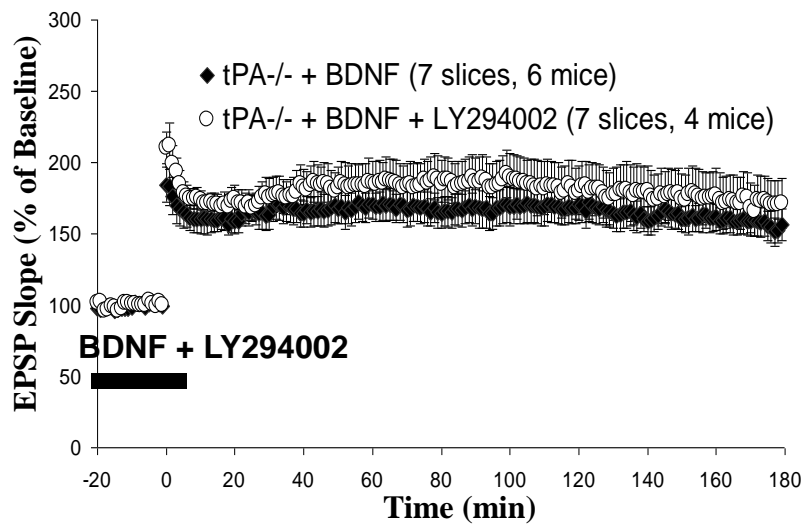**Supplementary Fig. 1**

Supplement: Supplementary Figure 1 [file npjscilearn20163-s1.pdf]

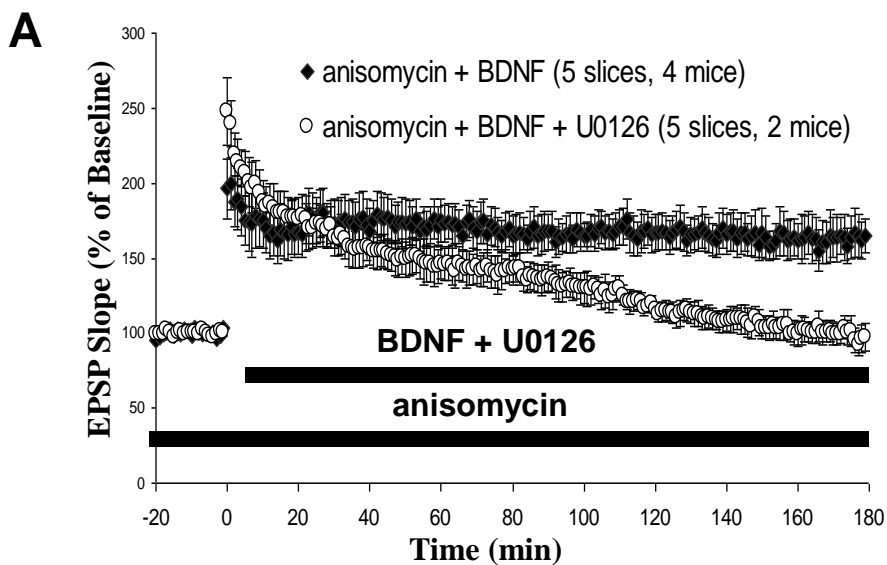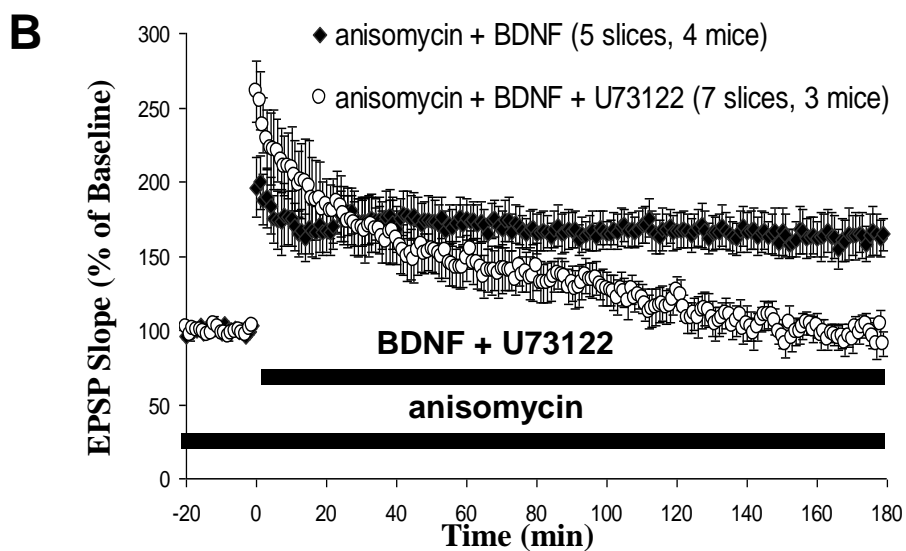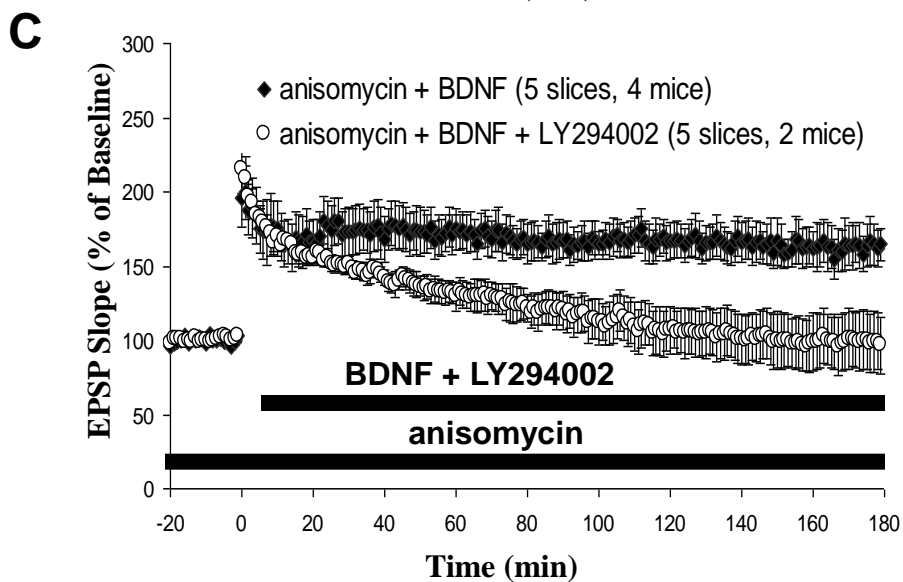

**Supplementary Fig. 2**

Supplement: Supplementary Figure 2 [file npjscilearn20163-s2.pdf]

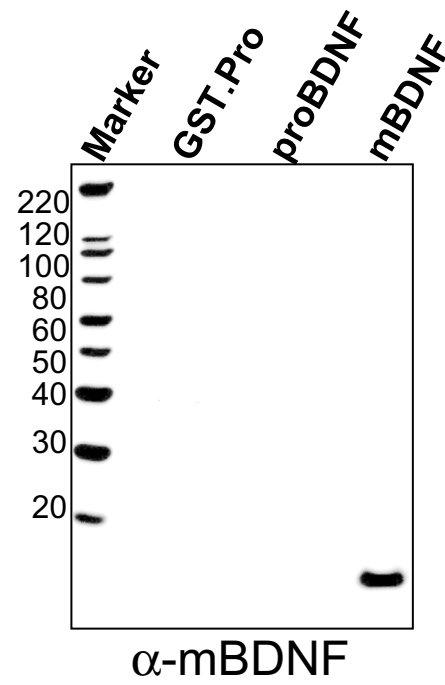

Supplemental Fig.3

Supplement: Supplementary Figure 3 [file npjscilearn20163-s3.pdf]

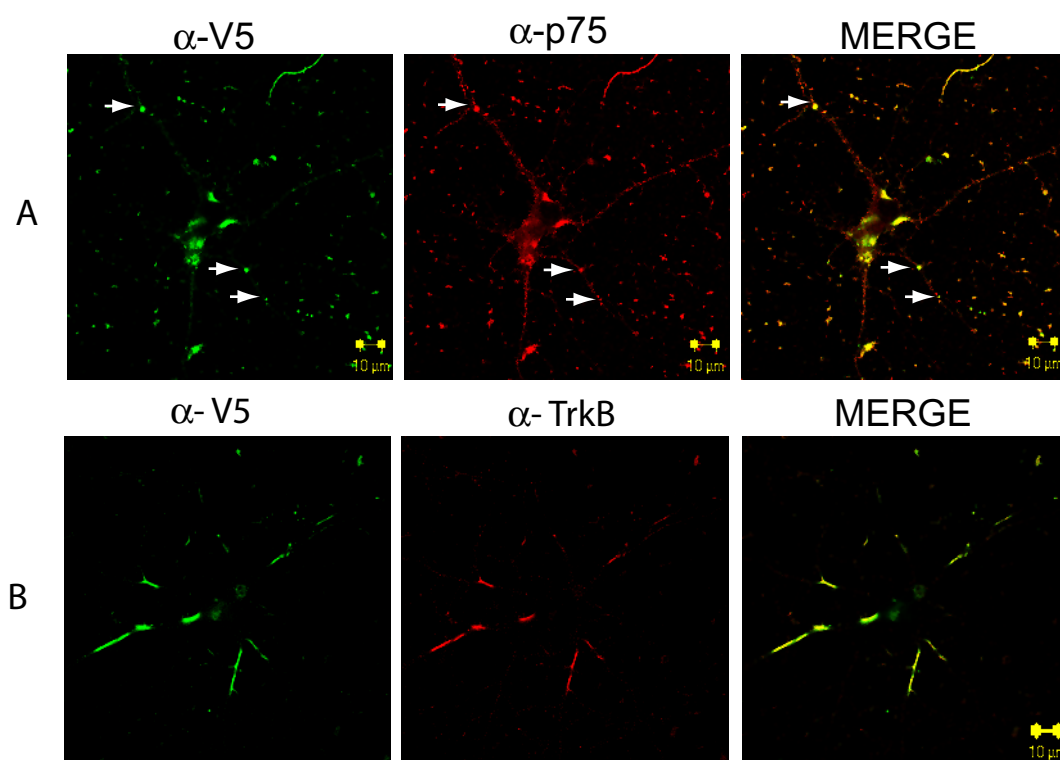

Supplemental Figure 4

Supplement: Supplementary Figure 4 [file npjscilearn20163-s4.pdf]
