# Supplementary material for: Extracellular and intracellular cleavages of proBDNF required at two distinct stages of late-phase LTP
Source: NPJ Sci Learn. 2016 May 11;1:16003. doi: 10.1038/npjscilearn.2016.3 (PMC6380376; doi:10.1038/npjscilearn.2016.3)
Supplement: Supplementary Information [file npjscilearn20163-s5.doc]

**Supplemental Figure 1. Signaling mechanisms underlying BDNF regulation at stage I.**

Hippocampal slices from tPA-/- mice were perfused with BDNF, together with indicated drugs, at stage I as indicated by the bars. (**A)** BDNF applied at stage I rescued the L-LTP deficit in tPA-/- slices, but simultaneous application of the MAPK inhibitor U0126 reversed the BDNF effect. (**B)** Inhibition of PLC-γ by U73122 did not alter the rescuing effect of BDNF on L-LTP deficit. (**C)** Inhibition of PI3K by LY294002 did not alter the rescuing effect of BDNF on L-LTP deficit.

**Supplemental Figure 2. Signaling mechanisms underlying BDNF regulation at stage II**

Wild type slices were perfused with anisomycin throughout the recording to block protein synthesis. BDNF, together with indicated drugs, was applied at stage II as indicated by the bars. Inhibition of MAPK by U0126 (**A**), PLC-γ by U73122 (**B**), or PI3K by LY294002 (**C**) all reversed the rescuing of BDNF on L-LTP deficit.

**Supplemental Figure 3. Western blot showing the specificity of anti-mBDNF antibody**

Equal amounts of purified proteins (GST fused to pro-domain of BDNF, uncleavable proBDNF and mBDNF) were resolved on NuPAGE, transferred to PVDF membrane, and probed using the affinity-purified anti-mBDNF antibody. Arrows point to the positions of the GST-Pro fragment and proBDNF, respectively. Note that the mBDNF antibody detects only mBDNF, and nothing else.

**Supplemental Figure 4. Co-localization of cell surface BDNF with p75^NTR^ or TrkB**

Hippocampal neurons (>14DIV) transfected with construct described in Fig. **5A** were fixed for non-permeable double staining using antibody against V5 (green, detect both proBDNF and mBDNF) and antibody against p75^NTR^ or TrkB. Note that majority of BDNF is co-localized with p75^NTR^ or TrkB on cell surface.
